# Stallion spermatozoa selected by single layer centrifugation are capable of fertilization after storage for up to 96 h at 6°C prior to artificial insemination

**DOI:** 10.1186/1751-0147-54-40

**Published:** 2012-07-12

**Authors:** Johanna Lindahl, Anne-Marie Dalin, Gesa Stuhtmann, Jane M Morrell

**Affiliations:** 1 Department of Reproduction, Clinical Sciences, Swedish University of Agricultural Sciences (SLU), Box 7054, 75007, Uppsala, Sweden; 2 Flyinge AB, 247 29, Flyinge, Sweden

**Keywords:** Single layer centrifugation, Stored stallion spermatozoa, Fertilizing capability, Artificial insemination

## Abstract

**Background:**

One of the challenges faced by equine breeders is ensuring delivery of good quality semen doses for artificial insemination when the mare is due to ovulate. Single Layer Centrifugation (SLC) has been shown to select morphologically normal spermatozoa with intact chromatin and good progressive motility from the rest of the ejaculate, and to prolong the life of these selected spermatozoa *in vitro*. The objective of the present study was a proof of concept, to determine whether fertilizing ability was retained in SLC-selected spermatozoa during prolonged storage.

**Findings:**

Sixteen mares were inseminated with SLC-selected sperm doses that had been cooled and stored at 6°C for 48 h, 72 h or 96 h. Embryos were identified in 11 mares by ultrasound examination 16–18 days after presumed ovulation.

**Conclusion:**

SLC-selected stallion spermatozoa stored for up to 96 h are capable of fertilization.

## Background

In Europe, the most usual method of equine artificial insemination (AI) is with cooled semen doses, with AI taking place within 24-36 h of semen collection. However, there can be problems with delivery of cooled semen on the day when it is required, particularly over the weekends and during public holidays. Although some stallion ejaculates will retain satisfactory sperm quality for 24-36 h [1], others do not tolerate cooling at all. The timing of AI relative to ovulation is problematic where mares are not stimulated to ovulate by the administration of hormones, which means that several AIs may be necessary in one oestrus to achieve conception. Therefore, a practical and efficacious method of prolonging the useable shelf-life of semen doses would be beneficial to the equine breeding industry.

Single Layer Centrifugation (SLC) is a colloid centrifugation technique designed to select spermatozoa with good motility, membrane integrity, normal morphology and intact chromatin from the rest of the ejaculate [2]. Progressive motility is prolonged in SLC-selected sperm samples, at least up to 72 h and often longer [3-5]. Since sperm morphology and chromatin integrity are improved in the SLC-selected sperm samples [6], it would be expected that these selected spermatozoa might retain their fertilizing ability over the same period.

## Objective

The present preliminary study was designed to test the **proof of concept** that SLC-selected spermatozoa from stallions at commercial AI studs retain fertilizing capacity when stored at 6°C for 48, 72 or 96 h after semen collection.

## Methods

### *Semen collection*

Semen was collected thrice weekly from stallions of known fertility (n = 6; age range 4 to 14 years, median 6 years) at commercial AI stations in Sweden according to standard husbandry practices. The sperm concentration of the raw ejaculate was estimated using a Spermacue (Minitube, Tiefenbach, Germany) and was adjusted to approximately 100 x10^6^/mL with warm (35°C) INRA96 (IMV, l´Aigle, France).

### *Preparation of SLC-selected sperm doses*

The colloid, Androcoll-E or Androcoll-E-Large (available from JM Morrell, SLU; patent applied for), 4 mL or 15 mL respectively, equilibrated to room temperature, was poured into 12 mL Sarstedt centrifuge tubes or 50 mL Falcon tubes [7]. After layering extended semen (4.5 mL or 15 mL) on top of the colloid, the tubes were centrifuged for 20 min at 300 g in a bench centrifuge with a swing-out rotor. The sperm pellet was harvested into a clean centrifuge tube containing fresh INRA96. Sperm concentration was measured with a Nucleocounter-SP100 (Chemometec, Denmark) [8] and was adjusted to approximately 50 x10^6^/mL, to prepare AI doses of 500x10^6^ where possible. These SLC-selected sperm doses were cooled to approximately 6°C and transported to SLU overnight in Styrofoam boxes containing a cold pack.

## Mare husbandry, oestrous detection and insemination

The mares (11 in total; average age 13 years, range 4–18 years) were kept at grass at the animal facilities of the Department of Reproduction, Swedish University of Agricultural Sciences. The study group included one maiden mare (number 16) and one mare that had foaled the year before the study (number 8). The reproductive history of the remaining mares was unknown although they were deemed to have normal oestrus cycles. The experiment took place in one oestrus cycle per mare at the beginning of two breeding seasons.

Ultrasound examination (Esaote Piemedical, Aquila^Pro^, 6 MHz probe) was carried out once daily until ovulation was judged to be imminent, when the frequency of examination was increased to twice daily. Inseminations were made when ovulation was expected within 12 hours, or if ovulation had already taken place. The criteria on which the decision to inseminate was based were the appearance of the dominant follicle (size, softness), the occurrance of uterine oedema, and signs of oestrus. Insemination was performed once only using a sterile uterine catheter and a dose of approximately 500x10^6^ spermatozoa, except for one mare where only 350 x10^6^ spermatozoa were available for AI. The mares were allocated randomly to the different treatment groups both with regard to the length of storage of the SLC-sample and to the stallion. After AI, the mares were examined by ultrasound every 6–24 hours until ovulation had occurred. The time of ovulation was estimated to occur between the last pre-ovulatory examination and the examination at which a luteal body was noted. Mares were examined again 16–18 days after the day of ovulation and the presence of an embryonic vesicle noted and measured. If no conceptus could be observed at the first examination, the mare was re-examined 2 days later. The mares were treated with prostaglandin (PGF2α) to terminate pregnancy.

All animal handling procedures were carried out according to standard husbandry and breeding practices. The experiment had been approved by the Ethics Committee.

## Statistics

The conception rates for AI at 48 h, 72 h and 96 h after semen collection were compared using the Chi-squared test.

## Results

**Figure 1:**
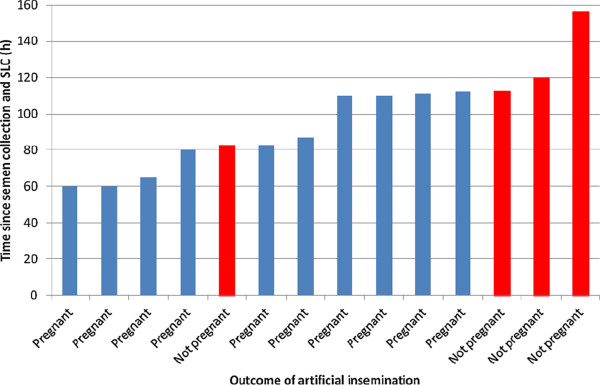
**Relationship between estimated sperm age at time of ovulation and outcome of artificial insemination (n = 14).** Note: the mares which were inseminated after ovulation have not been included in this diagram.

**Figure 2:**
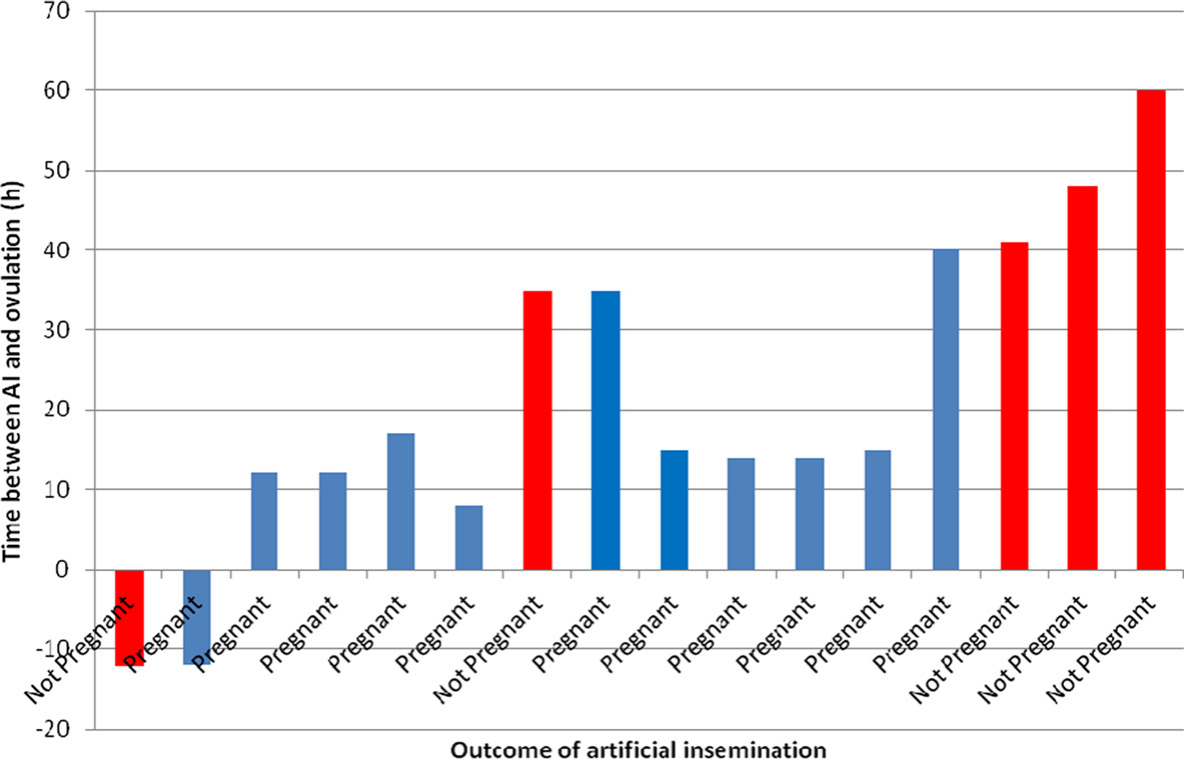
**Time between artificial insemination and ovulation and the outcome of the artificial insemination (n = 16).** Note: the data are sorted according to age of spermatozoa, as in Figure 1.

The presence of embryos in the uterus was detected by ultrasound in 11 of the 16 mares (69%) (Table 1). Conception rates were 80% (4/5) for 48 h-stored spermatozoa, 57% (4/7) for 72 h-stored sperm samples and 75% (3/4) for 96 h-stored sperm samples, which are not significantly different (P > 0.05). It was estimated that SLC-selected spermatozoa were capable of fertilization and subsequent oocyte activation for at least 110 h after SLC-preparation (Figure 1), although most of the AIs with spermatozoa >110 h coincidentally took place where there was a longer interval from AI to ovulation (Figure 2). The size of the embryonic vesicle was within the range 2.0-2.7cm, i.e. within the normal range for this period after conception.

**Table 1:** Outcome of artificial insemination with SLC-selected sperm doses stored for 48–96 hours (n = 16)

**Mare**	**Stallion**	**Time of AI after semen collection and SLC (h)**	**Sperm dose (x10**^**6**^**)**	**Conceptus detected by ultrasound**	**Age of spermatozoa at time of ovulation (h)**
1	B	48	500	Yes	65
2	D	48	500	Yes	60
3	F	48	500	No	83
4	F	48	500	Yes	83
5	F	48	500	Yes	60
6	A	72	500	No	113
7	A	72	500	No	AI after ovulation(< 12 h)
8	C	72	500	Yes	87
9	B	72	500	Yes	80
10	D	72	500	Yes	112
11	E	72	350**	Yes	AI after ovulation(< 12 h)
12	F	72	500	No	120
13	B	96	500	Yes (twins)	111
14	C	96	500	Yes	110
15	C	96	500	Yes	110
16	C	96	500	No	156

One additional mare, inseminated with semen from stallion A at 48 h after SLC, had already ovulated for more than12 h by the time of AI. She did not become pregnant, which may have been due to the interval between ovulation and AI.

** only 350 x10^6^ spermatozoa were available from stallion E.

## Discussion

The results presented here show that SLC-selected spermatozoa achieved fertilization and subsequent activation of the oocytes even after storage for up to 96 h at 6 °C. In fact, the SLC-selected spermatozoa fertilized oocytes up to an estimated 113 h after semen collection, as shown in Table 1. Two of the mares had already ovulated at the time of AI. Therefore, it is likely that they did not conceive because the oocytes would have been too old for fertilization by the time that spermatozoa were in the oviducts rather than because of a problem with the fertility of the SLC-sample. If these two mares are excluded from the total, the per cycle conception rate from SLC-selected sperm samples would be 11/14 or 79%. The per cycle conception rates for cooled sperm doses from these stallions, i.e. after AI at approximately 24 h after semen collection, and the number of mares, were as follows: stallion A 67% (24), stallion B 74% (23), stallion C 64% (97), stallion E 70% (10), stallion F 50% (20). The per season conception rate for Stallion D was 90% (the per cycle pregnancy rate is not available for this stallion).

Since the horses were kept at pasture, no examinations were carried out at night, when most ovulations actually occur [9]. Thus the exact timing of ovulation relative to insemination was not known. Even so, the number of pregnancies followed a pattern, with most pregnancies occurring when the spermatozoa were less than 113 hours old at the time of insemination, although the fact that most of the AIs with spermatozoa >110 h coincidentally took place where there was a longer interval from AI to ovulation could indicate that the lack of pregnancy was not necessarily due to the age of the SLC-selected spermatozoa. Pregnancy was terminated before day 20 to provide as accurate a picture as possible of the sperm effects on pregnancy. If the embryo was already dead at the time of ultrasound examination, the yolk sac would most likely have been smaller in size than observed [10]. The size of the embryonic vesicle was considered to be within the normal range for this time after AI [11,12]. Although waiting longer before termination would have provided more information about the normality of embryo development, it would also have allowed non-sperm factors to be introduced. Now that we have established the proof of concept that the spermatozoa are capable of fertilization up to 112 hours after semen collection and SLC-selection, it is our intention to perform additional studies in which subsequent embryo development, among other factors, will be studied.

Other studies with stored semen have shown that spermatozoa from the first jet of the ejaculate, extended in Kenney´s extender and used for insemination after 70 h and 80 h of storage using a dose of 2x10^9^ spermatozoa gave a pregnancy rate of 65% [13]. However, this method of collection requires an open-ended artificial vagina, which is not readily available on most studs, and a certain amount of skill to use the technique. Furthermore, the insemination dose was very large in that study, four times the dose used in the study described here; such large doses are not practical for a busy stud. Using standard collection methods, storage of semen extended in INRA96 for 48 h did not influence pregnancy rate negatively compared to fresh semen doses [14,15]. To our knowledge, this present study is the first to report conception rates after storing cooled SLC-selected sperm doses for long intervals (48-96 h) after semen collection. The stallions used for this trial were of known fertility and were selected at random from those available at the studs. In contrast, the reproductive histories of the mares were largely unknown and only one of them had carried a foal in the recent past. This might have had an adverse effect on the outcome of AI compared to using reproductively active mares of known fertility. However, it is interesting to note that satisfactory conception rates were achieved in such mares when SLC-selected spermatozoa were used. The pregnancies in this study were terminated at day 16–18. A full-scale AI trial with SLC-sperm doses subjected to prolonged cooled storage, in which the pregnancies are followed to their natural conclusion, is warranted to establish whether SLC could prolong the shelf-life of semen doses sufficiently to be useful for the equine breeding industry.

SLC has been used previously to obtain good quality sperm samples from problem stallion ejaculates [16]. However, early studies with Percoll™ (polyvinylpyrrolidone-coated silica particles) showed that although centrifugation with Percoll™ improved the quality of poor semen samples, it did not improve sperm quality in “normal” ejaculates [17]. The current study used Androcoll-E, a silica colloid optimized for stallion spermatozoa [4], which may explain why sperm quality was improved in SLC-selected samples from “normal” ejaculates. The SLC-technique is not difficult to perform and takes approximately 30 min per ejaculate, including the 20-min centrifugation, which would enable it to be incorporated into the stud´s normal semen processing routine. A fertility trial is currently underway to investigate whether the apparent improvement in sperm quality seen in laboratory assays in SLC-selected sperm samples compared to controls is reflected in an improvement in fertility when AI is performed at 24 h after semen collection, as is normally the case for cooled sperm doses.

In conclusion, SLC-selected sperm samples stored for 48-96 h at 6°C were capable of fertilization. The longest time interval between semen collection/SLC-processing and successful fertilization in this study was 112 h. Thus the proof of concept that SLC-selected spermatozoa are capable of fertilization after prolonged storage has been verified.

## Competing interest

Dr. Morrell has applied for a patent for Androcoll-E.

## Authors’ contributions

JL and A-MD carried out the cycle monitoring of the mares, did the inseminations and the pregnancy checks; JMM and GS prepared the SLC samples; the study was designed and organized by JMM, and the manuscript was written by JMM. All the authors have read and approved the manuscript.
